# Developing a primary care-initiated hepatitis C treatment pathway in Scotland: a qualitative study

**DOI:** 10.3399/BJGP.2022.0044

**Published:** 2022-05-24

**Authors:** David Whiteley, Elizabeth M Speakman, Lawrie Elliott, Helen Jarvis, Katherine Davidson, Michael Quinn, Paul Flowers

**Affiliations:** Department of Nursing and Community Health, School of Health and Life Sciences, Glasgow Caledonian University, Glasgow.; School of Health and Social Care, Edinburgh Napier University, Edinburgh.; Department of Nursing and Community Health, School of Health and Life Sciences, Glasgow Caledonian University, Glasgow.; Newcastle University, Newcastle; GP partner, the Bellingham Practice, Northumberland.; Department of Pharmacy, NHS Lothian, Edinburgh.; Craigmillar Medical Group, Edinburgh.; School of Psychological Sciences and Health, University of Strathclyde, Glasgow.

**Keywords:** general practice, hepatitis C, primary health care, qualitative research, Scotland, therapeutics

## Abstract

**Background:**

The ease of contemporary hepatitis C virus (HCV) therapy has prompted a global drive towards simplified and decentralised treatment pathways. In some countries, primary care has become an integral component of community-based HCV treatment provision. In the UK, however, the role of primary care providers remains largely focused on testing and diagnosis alone.

**Aim:**

To develop a primary care-initiated HCV treatment pathway for people who use drugs, and recommend theory-informed interventions to help embed that pathway into practice.

**Design and setting:**

A qualitative study informed by behaviour change theory. Semi-structured interviews were undertaken with key stakeholders (*n* = 38) primarily from two large conurbations in Scotland.

**Method:**

Analysis was three-stage. First, a broad pathway structure was outlined and then sequential pathway steps were specified; second, thematic data were aligned to pathway steps, and significant barriers and enablers were identified; and, third, the Theoretical Domains Framework and Behaviour Change Wheel were employed to systematically develop ideas to enhance pathway implementation, which stakeholders then appraised.

**Results:**

The proposed pathway structure spans broad, overarching challenges to primary care-initiated HCV treatment. The theory-informed recommendations align with influences on different behaviours at key pathway steps, and focus on relationship building, routinisation, education, combating stigmas, publicising the pathway, and treatment protocol development.

**Conclusion:**

This study provides the first practicable pathway for primary care-initiated HCV treatment in Scotland, and provides recommendations for wider implementation in the UK. It positions primary care providers as an integral part of community-based HCV treatment, providing workable solutions to ingrained barriers to care.

## INTRODUCTION

An estimated 58 million people were living with the hepatitis C virus (HCV) in 2019,^1^ equating to approximately 0.7% of the global population. In the UK, recent estimates suggest 118 000 individuals are chronically infected with HCV, with injecting drug use the most important risk factor for acquisition.^2^ While the number of people accessing HCV treatment is rising, the rate of increase has slowed in recent years, suggesting it is becoming harder to find, diagnose, and treat people living with HCV as the pool of infection gradually decreases.^2^

Historically, HCV treatment presented an arduous and challenging ordeal. Suboptimal toxic medications offered limited success and necessitated intensive monitoring and support.^3^ As such, these drugs were the sole domain of specialist practitioners in secondary and centralised care. Over the last decade a sea change has occurred, with a barrage of novel drugs coming to market that target specific steps within the HCV life cycle. Collectively, these direct-acting antivirals (DAAs) now offer a safe, simple, and effective cure with just 8–12 weeks of treatment.^4^ The relative ease of contemporary HCV drug therapy has spurred a global drive towards decentralisation, expanding access by relocating the nexus of care firmly within the community. The World Health Organization (WHO) endorses this move, calling for simplified and streamlined HCV treatment pathways to be integrated into existing healthcare systems, producing a plurality of community-based provision.^5^

Globally, this push for decentralised care has encouraged a raft of HCV treatment pathways in diverse settings, including harm reduction services, prisons, community pharmacies, and homeless facilities, as well as within general practice. Such pathways have repeatedly demonstrated increased uptake and comparable cure rates with treatment initiated in more traditional hospital-based settings, illustrating their viability and acceptability as loci of care.^6^^,^^7^ While many community-based treatment pathways continue to rely on specialist practitioners to initiate therapy (in an outreach model of provision), some have prioritised partial or comprehensive task-shifting to non-specialists, with no reduction in treatment efficacy.^8^ The importance of some degree of task-shifting is underpinned by arguments for enabling access in areas of inadequate healthcare infrastructure,^9^ and concerns that numbers of existing specialists are limited.^10^ Task-shifting is also supported by recent challenges to ‘medical speciality protectionism’ voiced by the European Association for the Study of the Liver.^11^

**Table 1:** How this fits in

Historically, GPs were rarely involved in the treatment of HCV, their role being more commonly restricted to viral testing and diagnosis. Contemporary drug therapy for HCV has allowed reconsideration of this status quo, and offers potential for GPsto initiate HCV treatment in primary care. This study provides a way forward, detailing a practicable theory-informed pathway and recommendations for primary care-initiated HCV treatment in the UK.

In the UK, while primary care has witnessed effective interventions to increase HCV testing and diagnosis,^12^ the role of most GPs in HCV treatment remains limited to specialist referral, or hosting specialist-led outreach clinics. Linkage from diagnosis to treatment is a known bottleneck in the HCV care cascade,^13^ and, while other countries have helped alleviate barriers to HCV treatment by more comprehensive integration of GPs into models of DAA provision,^14^ the UK is yet to follow suit. The study authors have previously identified overarching challenges to primary care provision of HCV treatment in Scotland,^15^ and here this work is moved forward by articulating what a practicable, acceptable, and sustainable model of primary care-initiated HCV treatment could look like. The aim is to develop a primary care-initiated HCV treatment pathway for the UK, and recommend theory-informed intervention elements to embed that pathway into practice.

## METHOD

The research team united academics and clinicians, offering a blend of expertise and perspectives in HCV, general practice, behaviour change theory, and research methodology. The principal investigator is an early-career researcher and former HCV nurse specialist, supported by a professor of public health and a professor of psychology. The rest of the team comprises experienced clinicians working in general practice and secondary care, and an experienced research fellow.

### Theoretical framework

Increasing evidence suggests public health interventions grounded in theory may be more effective than those that lack a theoretical basis.^16^ Therefore, the implementation of primary care-initiated HCV treatment was conceptualised as requiring the development of a complex multi-actor/agent behaviour change intervention. It asks which behaviours need to change, where and by whom they are being performed, and how these behaviours interact.^17^ To avoid constraints imposed by any single behavioural theory, the Theoretical Domains Framework (TDF) was employed to explore cognitive, affective, social, and environmental influences on behaviour. The TDF is an integrated meta-theoretical framework that combines 33 theories of behaviour or behaviour change into 14 domains and 84 theoretical constructs.^18^ It aims to make identification of the determinants of implementation behaviours more comprehensible, and is now widely used in intervention development studies, particularly within primary health care in developed nations.^19^ The TDF has also established links with the Behaviour Change Wheel (BCW), a framework for classifying the *types* of intervention required to effectively alter specific behaviours.^20^ Both the TDF and the BCW are employed within this study.

### Participants and setting

The concept of primary care-initiated HCV treatment has implications for diverse stakeholders, and therefore a purposive maximum variation sample was used. This comprised:

- GPs currently providing care for people who use drugs;
- people who use, or have used drugs, and are living with HCV (hereafter referred to as people living with HCV [PLHCV]);
- HCV specialists (doctors, nurses, and pharmacists) working in hepatology and/or infectious diseases;
- staff from agencies providing support for PLHCV; and
- representatives from community pharmacies and NHS procurement.

The study was primarily located within two Scottish health boards with the highest number of new HCV diagnoses in 2018,^21^ although recruitment of GPs widened to six additional Scottish health boards following study inception. The impact of the COVID-19 pandemic meant data collection was suspended in March 2020, with the study subsequently suspended in its entirety from May until August 2020.

### Recruitment

GPs were recruited in two ways. First, an advertisement was placed in a monthly newsletter sent to all GP practices by the NHS Research Scotland Primary Care Network, inviting GPs to contact the study team. Second, GP practices within areas of social deprivation in two NHS boards were identified and invited to participate using publicly available email addresses. HCV specialists and community pharmacists were recruited through the existing networks of two authors, who purposively identified key individuals. Support agencies and NHS procurement were also approached directly, and the most appropriate potential participants within these organisations were collaboratively identified. Interested responders from all groups were given further information about the study and time to consider their involvement before interviews were arranged at a time and place convenient for the interviewee.

Recruitment of PLHCV was led by the Scottish Drugs Forum (SDF), who work with and support a wide network of people who use drugs, many of whom are PLHCV. Staff from SDF approached PLHCV, who, if interested, were provided with further details of the study and given time to consider participation. Other avenues of recruitment for PLHCV were abandoned early in the study, and before any participants were recruited, to prevent additional pressure on NHS services during the COVID-19 pandemic.

### Data generation

Data were generated through semi-structured interviews. Two phases of interviews were compelled by the study suspension, with the first phase between October 2019 and March 2020, and the second phase between October 2020 and January 2021. Interviews with care providers were predominantly conducted in person during phase one, and using teleconferencing software in phase two. Interviews with PLHCV took place exclusively during phase two and were conducted remotely by SDF peer researchers, all of whom had lived experience of drug use and HCV. These interviews were facilitated by a member of SDF staff during a three-way conference call. Topic guides, specific to each participant group, were used to focus conversation. The topic guide for PLHCV was revised in consultation with SDF peer researchers before use. In addition, examples of published primary care-initiated HCV treatment pathways were used as stimulus material in phase one interviews,^22^^,^^23^ with the developing pathway also presented for discussion during interviews in phase two (that is, key steps of the pathway helped to focus data generation). All interviews were audio-recorded, transcribed verbatim, and pseudonymised. Finally, participants were asked if they would be willing to participate in a further focus group at a later date.

### Data analysis: pathway development

**Figure 1.**
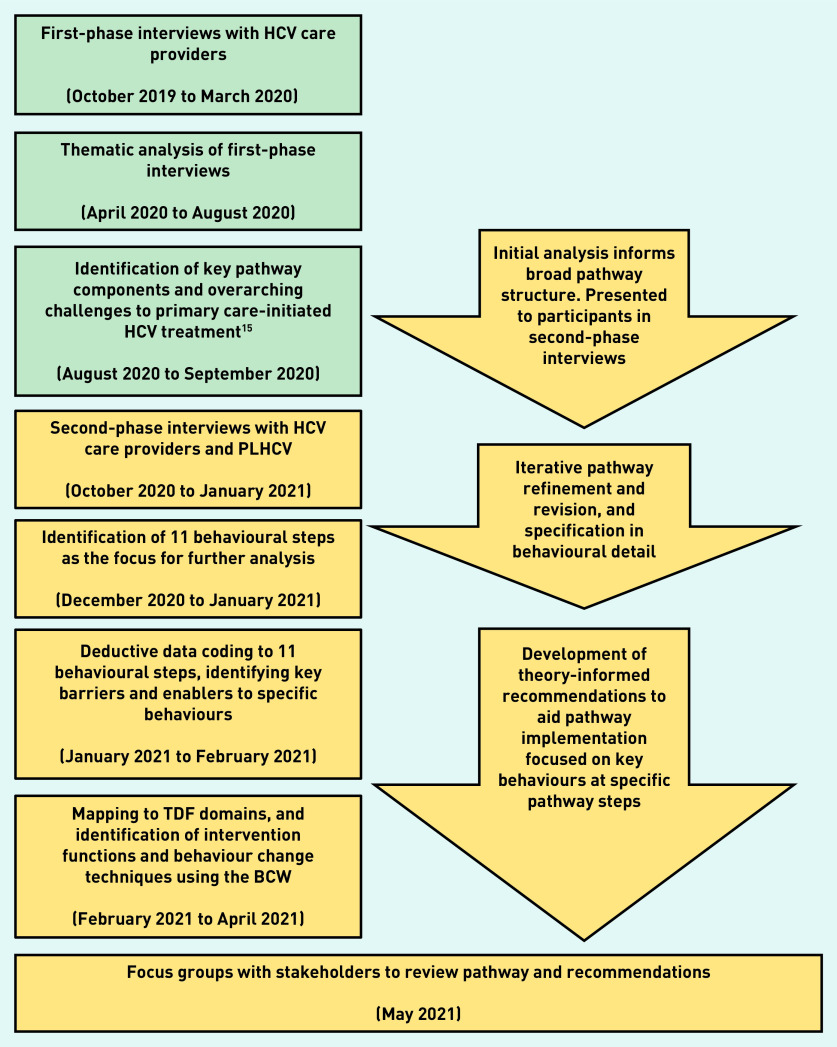
***Sequential depiction of methodology, alongside pathway and recommendation development.****^a^ ^a^****Stages in green have been described previously.****^15^* ***BCW = Behaviour Change Wheel. HCV = hepatitis C virus. PLHCV = people living with HCV. TDF = Theoretical Domains Framework.***

**Figure 2.**
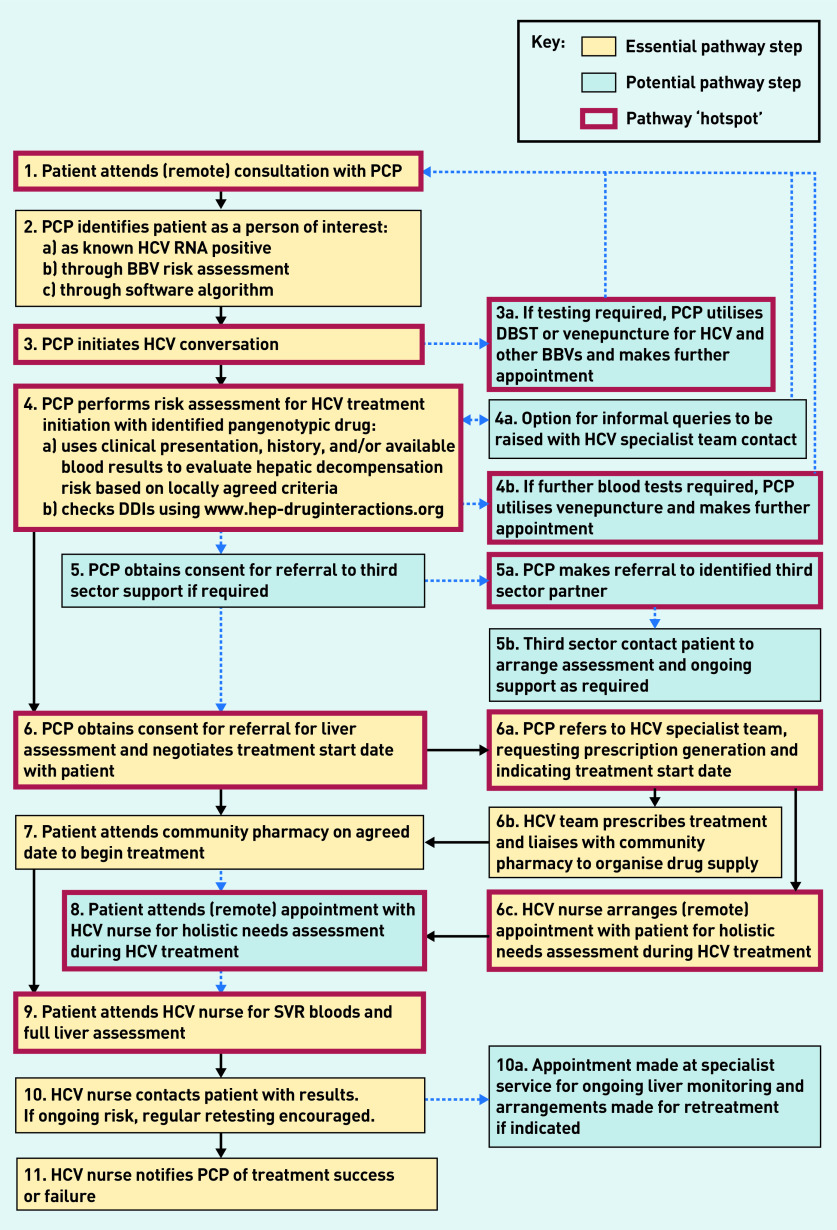
*A pragmatic primary care-initiated HCV treatment pathway. BBV = blood-borne virus. DBST = dried blood spot testing. DDI = drug–drug interactions. HCV = hepatitis C virus. PCP = primary care provider. RNA = ribonucleic acid. SVR = sustained virological response.*

Initially, data from first phase interviews with care providers were thematically analysed to identify fundamental components of a primary care-initiated HCV treatment pathway, and to explore broad, overarching challenges to its success. This stage of analysis has been described in detail elsewhere, and findings previously reported.^15^ For clarity, Figure 1 illustrates where this preliminary work sits within the process of pathway and recommendation development described here. An initial, simple pathway structure grew from this early analysis, which was iteratively revised, refined, and expanded during phase two interviews in light of emerging insights from the data. During this time, four authors met regularly to interrogate the developing pathway and specify the component parts as a series of behavioural steps, detailing *who* was enacting *what* behaviour at each individual point. Individual steps were further specified as ‘essential’ or ‘potential’, and the evolving pathway regularly examined and critiqued by the wider study team, challenging its logic and lucidity. Eleven steps within the pathway were then collaboratively identified that either significantly diverged from routine clinical practice or were deemed ‘hotspots’ critical to success. In this way, key elements of a potential pathway were iteratively developed. This stage ended with a clear set of sequential behavioural steps that together provided the basic elements of a potential primary care pathway for HCV treatment (Figure 2). Subsequent analysis fleshed out how these steps could be operationalised.

### Data analysis: recommendation development

The behavioural steps specified within the pathway comprised focal points for recommendation development to make the pathway work and be fit for purpose. Using a coding framework, two authors deductively coded relevant data to each of the 11 steps, identified key barriers and facilitators at each point, and ranked them reflecting their prominence within the data. NVivo (version 12) software was used to manage this process. These barriers and facilitators were then mapped to domains of the TDF, to categorise influences on the stated behaviour. Next, established links between the TDF and BCW were utilised to identify precisely *how* these influences could be changed. Each domain of the TDF aligns with specific intervention functions (that is, *how* interventions might change behaviours), which link to a taxonomy of behaviour change techniques.^17^ For example, if the data suggested a knowledge deficit represented a key barrier at a particular pathway step, this would be coded to the theoretical domain *‘knowledge’* using the TDF, the BCW would then suggest the intervention function *‘education’*, which could be operationalised with the behaviour change technique *‘providing instructions on how to perform a behaviour’*. This process was regularly audited by a Health and Care Professions Council Registered Health Psychologist, who interrogated the initial TDF mapping, and provided robust review of the alignment to intervention functions and classified behaviour change techniques. From here, through robust and repeated discussions, the research team collaboratively developed detailed recommendations that specified how the pathway could be implemented. This stage ended with a rich set of theoretically informed and evidence-based recommendations for how to operationalise the proposed pathway. Subsequent work made sure these were pragmatic and likely to be sustainable.

### Data analysis: stakeholder review

Three focus groups were convened in May 2021 to provide expert review of both pathway and recommendations, with a particular focus on whether they could be delivered and were sustainable within current provision. Participants who had previously expressed interest were approached, and a self-selecting sample asked to participate in one of three 2-hour online focus groups, facilitated by two authors using teleconferencing software. The APEASE criteria (Affordability, Practicability, Effectiveness, Acceptability, Side-effects, and Equity)^17^ were used to guide discussion, with participants asked to reject, modify, or accept the proposed recommendations. Focus groups were audio-recorded for reference, and any subsequent modifications made to the pathway and/or recommendations were reviewed by the study team.

## RESULTS

Thirty-eight participants were interviewed. They comprised 11 GPs, 10 PLHCV, nine HCV specialists (four consultants, three specialist nurses, and two specialist pharmacists), five staff from agencies providing support for PLHCV, two individuals employed by NHS procurement with insight into DAA reimbursement, and one community pharmacist. Twelve participants subsequently took part in one of the three focus groups, which comprised six GPs, two PLHCV, two HCV specialists (one consultant and one specialist pharmacist), and two staff from HCV support agencies.

### Pathway

Figure 2 shows the final pathway developed, with 20 individual behavioural steps detailing *who* needs to do *what* within each sequential element. It expands on the basic building blocks of DAA treatment, but maintains three essential elements: the provision of drug (steps 6, 6a, 6b, and 7), the assessment of liver fibrosis (steps 6 and 9), and opportunities for the provision of holistic support (steps 5, 5a, 5b, 6c, and 8).^15^ While GPs were the initial focus of this study, the more generic term primary care provider (PCP) has been purposefully used within the pathway, recognising that GPs are not the only practitioners located in GP surgeries who could initiate HCV treatment in primary care. Figure 2 also distinguishes steps characterised as ‘essential’ or ‘potential’ and shows which were designated ‘hotspots’ in particular need of operationalisation. These eleven hotspots incorporated both essential and potential steps, and covered the patient journey from attending an initial appointment with a PCP, to a post-treatment test of cure and liver fibrosis assessment. Steps 3a and 4b both focused on the utilisation of dried blood spot testing and/or venepuncture by PCPs, and were therefore combined within the analysis.

### Detailed recommendations for pathway implementation

While pathway structure could help alleviate broad, overarching challenges previously identified,^15^ the behavioural specification of the pathway allowed specific barriers and facilitators to individual actions at each hotspot to be addressed. Supplementary Box S1 provides an overview of 21 key barriers and facilitators identified across the 11 pathway hotspots, alongside illustrative quotes. The identified barriers and facilitators related to multiple behaviours by different actors across the pathway stages. An overview of the final recommendations is provided in Supplementary Box S2, detailed by each pathway step, following the TDF and BCW analysis and subsequent stakeholder appraisal. Of the 82 interventions originally suggested, stakeholders rejected 25, and modified eight of the remaining 57.

Although diverse, the suggested recommendations could be categorised into one of six broad areas of intervention. The first area of intervention was designed to foster and build professional and therapeutic relationships, for example, prioritising continuity of carer for PLHCV, developing and utilising GP clusters and managed care networks, and creating connections between GP surgeries and third-sector partners. The second interventions were designed to help routinise and habituate the pathway into PCPs’ everyday practice. Included here were prompts within existing IT systems, and the establishment of clear and simple communication channels between PCPs and HCV specialist services. The third area of intervention was education. Specifically, this was training for PCPs on the pathway, HCV care, and the benefits of dried blood spot testing, alongside optimising opportunities to educate PLHCV about the advantages of pathway engagement. Here, informal education was key, with important roles for the third sector, peer support workers, and community link workers engaging in recurrent *ad hoc* conversations with PLHCV. The fourth interventions were designed to reduce the impact of social stigmas on pathway engagement, including offering HCV treatment within routine clinics, rather than identifiable ‘HCV clinics’ running on specific days and times. The fifth area was a focus on interventions to publicise the existence of the pathway to PLHCV, promoted by third-sector partners and other health and social care professionals embedded within the community. Finally, interventions were designed to protocolise the pathway into local contexts, including plans for audit and review. For the interested reader, full details of the analyses that underpin the findings are provided in Supplementary Box S3.

## DISCUSSION

### Summary

The decentralisation of HCV treatment into the community is a fundamental component of global and national HCV elimination plans.^2^^,^^5^ In some countries, GPs have been an integral cog in the decentralisation wheel for a number of years,^14^ but, in the UK, optimising the GP role has to date received little attention. To the authors’ knowledge, this study is the first to provide a tangible way forward, offering a detailed pathway for HCV treatment initiation by GPs and other PCPs in the UK. The pathway tackles previously identified challenges to primary care-initiated HCV treatment,^15^ while offering theory-informed recommendations to help overcome specific behavioural barriers, and embed the pathway into practice.

### Strengths and limitations

The study has a number of strengths. Participants were drawn from diverse stakeholders, offering contrasting perspectives and insights from both service providers and service users. Also, while the study suspension owing to COVID-19 was unplanned, it enhanced the iterative nature of pathway development by offering time for in-depth engagement with phase one interviews. Finally, effective public health initiatives are grounded in an understanding of health behaviours and the contexts in which they occur.^16^ By defining the pathway and recommendations in behavioural terms, and utilising established tools that translate behavioural theory into actionable recommendations (including wide stakeholder engagement), this study bridges the divide between theoretical and applied research.

There are also a number of limitations. The participants were a self-selecting sample, who may represent a particularly motivated group of individuals. The COVID-19 pandemic and associated lockdowns arrived in Scotland 6 months after the start of recruitment. This impacted recruitment and data generation options, particularly for PLHCV, who were entirely drawn from people engaging with SDF services, and who had the means to participate in a remote interview. Finally, it should be borne in mind that the pathway and recommendations also offer a defined medical endpoint, largely ignoring the post-cure lives of PLHCV.^24^

### Comparison with existing literature

WHO have called for the elimination of viral hepatitis as a major threat to global public health by 2030.^25^ Such an ambitious goal requires substantial increases in the testing and diagnosis of HCV, and innovative reassessment of where and how treatment can be provided.^26^ The arrival of DAAs has encouraged creative revision of HCV models of care, aimed at broadening access and simplifying the patient journey by decentralising care into the community, integrating with existing services, and task-shifting to less specialised healthcare workers.^9^ Of paramount importance is that these simplified models of care *work*.^6^^–^^8^

Previous studies have focused on identifying barriers and enablers to GP involvement in decentralised HCV treatment,^27^^–^^31^ but often abstain from identifying how such barriers can be addressed. This study offers a practical way forward. Ingrained barriers such as the pre-treatment assessment of liver fibrosis are removed, supported by a growing evidence base questioning its necessity.^32^ The frequently cited difficulties of venepuncture are sidestepped by the use of sensitive, specific, and accurate dried blood spot testing technologies.^33^ More problematic is the ability of PCPs to prescribe DAAs. While WHO recognises good practice in Scotland in coordinating primary and secondary care prescribing, and in medicine reimbursement policies,^34^ current constraints in relation to DAAs prevent PCPs prescribing the drugs. A solution to the convoluted process of DAA reimbursement and prescribing would allow further simplification of the pathway proposed here.

Scotland aims to exceed WHO targets and achieve HCV elimination by 2024 at the latest.^35^ To date, the primary focus of Scottish decentralised HCV treatment has been the co-location of outreach clinics within existing drug services, with limited task-shifting away from specialist clinicians.^36^ As self-imposed deadlines for HCV elimination loom, general practice and primary care remain peripheral to, or entirely absent from, the vision for community-based HCV treatment in Scotland.^35^ This is echoed throughout the UK, with the latest reports on progress towards HCV elimination from England and Wales locating the GP role clearly within HCV testing and diagnosis alone.^2^^,^^37^ The potential significance of omitting the contribution GPs could make to DAA prescribing is illustrated by comparison with other developed nations, notably Australia.^38^

Globally, Australia is a bellwether for HCV elimination, leading the charge in the decentralisation of HCV treatment from hospital to primary care. Most people now treated for HCV in Australia are prescribed DAAs by non-specialists in community settings.^38^ This fundamental change in disease management was catalysed by the removal of prescribing restrictions for DAAs in March 2016, enabling GPs to evolve their roles from test and refer, to test and treat. This move broadened access to DAAs and helped facilitate a rapid increase in HCV treatment uptake over the first 10 months.^39^ More recent data demonstrate that initial momentum has been sustained, with the number of GPs prescribing HCV treatment continuing to increase.^14^ While not without its challenges, the inclusion of GPs within models of decentralised HCV treatment expands access to people who use (or crucially *used*) drugs and are living with HCV but who do *not* engage with drug and alcohol services.^30^ While transplanting an Australian model of care into the UK may be tempting, doing so would ignore contextual factors that may limit its success, not least the complex system of drug reimbursement currently in operation for DAAs. A bespoke pathway, sensitive to local contexts, is required.

GP locality is also an important consideration. Scotland is a geographically diverse nation, with 30% of the population living in rural areas.^40^ The current focus of HCV treatment provision within drug services ignores the geographic inequity of remote and rural populations experiencing poorer access to health services than their urban counterparts.^40^ Recent drug death figures emphasise that people who use drugs in Scotland are not confined to urban settings,^41^ and historic data reporting HCV antibody prevalence suggest neither is HCV.^42^ For rural and remote communities, the role of the GP can differ from their urban counterparts, providing additional services and offering lifelong care to their communities from ‘cradle to grave’.^40^^,^^43^ Within this context, a pathway for primary care-initiated HCV treatment may provide the only feasible access to care for some PLHCV.

While this study advocates for further inclusion of GPs within the expanding web of community-based HCV treatment provision, it does so with a note of caution. This study sits within an ongoing recruitment crisis in general practice, which has only been exacerbated by the continuing COVID-19 pandemic.^44^^,^^45^ While it may be feasible and realisable for GPs to initiate HCV treatment with this pathway, the current strains on primary care provision may preclude its enthusiastic embrace.

### Implications for research and practice

Current HCV elimination policy in Scotland and the UK restricts the role of GPs to testing, diagnosis, and referral. To the authors’ knowledge, this study provides the first practicable pathway for primary care-initiated HCV treatment in the UK, offering GPs and other PCPs an opportunity to remove barriers to care and improve patient outcomes. Underpinned by theory, the pathway and associated recommendations offer a robust and realisable way to integrate primary care into the growing network of decentralised, community-based HCV treatment hubs.

Fifty-seven recommendations were identified to aid pathway implementation within six broad areas of intervention covering 11 pathway steps. These recommendations should not be taken as inflexible dogma, but as a toolkit to help adapt the pathway to different local contexts. For example, not all PCPs will need HCV training, and some GP practices will already have robust relationships with third-sector partners and local HCV specialist teams. The recommendations highlight focal points within the pathway that need to be considered, rather than the prescription of a one-size-fits-all solution. Future research should focus on the operationality and sustainability of the pathway through piloting.
